# Cardioprotective effect of aqueous extract of *Chichorium intybus* on ischemia-reperfusion injury in isolated rat heart

**Published:** 2015

**Authors:** Najmeh Sadeghi, Mahin Dianat, Mohammad Badavi, Ahad Malekzadeh

**Affiliations:** 1 *Department of Physiology, Faculty of Medicine, Jahrom University of Medical Sciences, Jahrom, Iran*; 2 *Physiology Research Center, Department of Physiology, Faculty of Medicine, Ahvaz Jundishapur University of Medical Sciences, Ahvaz, Iran*; 3 *Department of Statistic, Khajeh Nasir Toosi University of Technology, Tehran, Iran*

**Keywords:** *Chichorium intybus L.*, *Ischemia-Reperfusion*, *Heart Contractility*, *Rat*

## Abstract

**Objective:**

Several studies have shown that *Chichorium intybus* (*C. intybus*) which possesses flavonoid compounds has an effective role in treatment of cardiovascular diseases. Contractile dysfunction mostly occurs after acute myocardial infarction, cardiac bypass surgery, heart transplantation and coronary angioplasty.

The aim of the present study was to investigate the effect of aqueous extract of *C. intybus *on ischemia- reperfusion injury in isolated rat heart.

**Materials and Methods:**

The animals were divided into four groups (Sham, Control, 1 mg/ml and 3 mg/ml of extract) of 8 rats. The aorta was cannulated, and then the heart was mounted on a Langendorff apparatus. Next, a balloon was inserted into the left ventricle (LV) and peak positive value of time derivate of LV pressure (+dp/dt), coronary flow (CF), and left ventricular systolic pressure (LVSP) in pre-ischemia and reperfusion period were calculated by a Power Lab system. All groups underwent a 30-minute global ischemia followed by a 60-minute reperfusion.

**Results:**

The results showed that heart rate (HR), coronary flow, and left ventricular developed pressure (LVDP) and rate of pressure product (RPP) significantly decreased in the control group during reperfusion, while these values in the groups receiving the extract (3mg/ml) improved significantly during reperfusion (p<0.001).

**Conclusion:**

It seems that flavonoid compounds of aqueous extract of *C. intybus *reduce ischemia - reperfusion injuries, suggesting its protective effect on heart function after ischemia.

## Introduction

More than 3.5 million men and 3.4 million women die because of heart diseases each year. Coronary heart disease is one of the main causes of death among cardiovascular diseases in the world (Yellon and Hausenloy, 2007 ▶). Contractile dysfunction mostly occurs after acute myocardial infarction, cardiac bypass surgery, heart transplantation and coronary angioplasty (Bolli, 1992 ▶). 

When the supply of oxygen is disrupted as in the case of myocardial ischemia, mitochondrial electron transport chain (ETC) flux and oxidative phosphorylation (OXPHOS) decline, the pool of high energy phosphates is rapidly depleted, pyruvate oxidation decreases and ATP production is impaired (Marin-Garcia and Goldenthal, 2004 ▶; Kocki et al., 2006 ▶; Swiader et al., 2006 ▶).

After an acute myocardial infarction, early and successful myocardial reperfusion by thrombolytic therapy or primary percutaneous coronary intervention (PCI) is the most effective strategy for reducing the size of a myocardial infarct and improving the clinical outcome. The process of restoring blood flow to the ischemic myocardium can induce injury. This phenomenon, termed as myocardial reperfusion injury, can paradoxically reduce the beneficial effects of myocardial reperfusion (Banach et al., 2006 ▶; Yellon and Hausenloy, 2007 ▶). 

The perception of myocardial cell injury occurring after ischemia/reperfusion (I/R) involves two major hypotheses: increases in intracellular calcium and/or the accumulation of reactive oxygen species (ROS) causing the sarcolemmal peroxidation of the cellular phospholipid layer, leading to loss of cellular integrity and facilitating calcium entry (Marin-Garcia and Goldenthal, 2004 ▶; Piechowiak et al., 2006 ▶).

In addition, ROS production can cause extensive damage such as myocardial stunning. Previous studies have shown that red wine extract and antioxidant treatment significantly improved post-ischemic ventricular and contractile function (Das et al., 1999 ▶; Sato et al., 2000 ▶).

To reduce lethal reperfusion injury, new cardioprotective strategies have been applied. These include preconditioning with antioxidants, activators of the reperfusion injury salvage kinase (RISK) pathway, inhibitors of protein kinase c-delta, and inhibitors of the mitochondrial permeability transition pore mPTP (Buja and Weerasinghe, 2010 ▶).

It has been proved that eating fresh vegetables and fruits can prevent cardiovascular diseases (CVD) (Argolo et al., 2004 ▶) due to the presence of natural antioxidants such as flavonoid, anthocyanin and phenolic compounds (Zhang and Wang, 2002 ▶). Previous studies showed that *Chichorium intybus* L. which has flavonoid compounds has an effective role in treatment of many diseases. For example, a study has reported that *C. intybus* extract protects the liver against thioacetamide-induced hepatotoxicity in rats (Madani et al., 2006 ▶). Other researchers found that Chichorium decreases blood glucose in rats fed with diets containing chiycoryina (Khaksari et al., 2001 ▶). Others reported that *C. intybus *decreases calcium influx from the extra cellular space and induces vasorelaxation in isolated rat aorta strips (Sakurai et al., 2003 ▶). Also, prior studies have shown that a diet containing *C. intybus *can decrease the pH and viscosity in the colon and secum in rabbits and rats due to inhibition of the enzymes’ activity and decrement in the Triglyceride (TG), cholesterol and the blood sugar in diabetic rats (16, 10, and 12). 

An extensive literature survey has shown that there are no scientific reports available on the effect of *C. intybus* on hemodynamic factors after I/R injury. Therefore, the aim of the present study was to investigate the effect of aqueous extract of *C. intybus *on ischemia- reperfusion injury in isolated heart of rats, because it has not been investigated yet. The results of this study could reduce the excessive costs of I/R injury.

## Materials and Methods

**Animals**

Male Wistar rats (body weight 250 -300 g) were used in this study. The animals were divided randomly into the following groups of 8 rats: Sham, Control, CH1 (1mg/ml *C. intybus* extract) and CH2 (3mg/ml *C. intybus *extract). ( Roohbakhsh and Karimi, 2009 ▶;). *C. intybus *extract was dissolved in perfusate solution and administered from the beginning of reperfusion and maintained in the perfusion buffer (Krebs-Henseleit bicarbonate) during the 60-minute period of reperfusion ( Roohbakhsh and Karimi, 2009 ▶).

**Drugs**

Heparin and triphenyl tetrazolium chloride (TTC) were purchased from Sigma (St. Louis, MO). Sodium chloride, potassium chloride, magnesium sulphate, sodium hydrogen carbonate, potassium hydrogen orthophosphate, D-glucose and calcium chloride were obtained from Merck Laboratories. Ketamine and xylazine were purchased from Alfasan Co (Woderen- Holland).

**Preparation of the aqueous extract**

*C. intybus *was obtained from market, Ahvaz, Iran, in the summer and identified by herbalists of Ferdwosi University, Mashhad, Iran. Then, it was dried in a dry and dark place. After that, it was grinned to prepare the aqueous extract. Finally, 10-15 gr of this flour was soaked in 200 ml distilled water for 3 days. Afterwards, it was filtrated and evaporated at 40-45 ˚C in the water bath (Roohbakhsh and Karimi, 2009 ▶).

**Experimental protocol**

Rats were anesthetized using an intra-peritoneal (IP) injection of ketamine HCl (50 mg/kg), xylazine (5 mg/kg); heparin (1000 U/kg) was also administered (IP). The trachea was cannulated and the rats were ventilated by room air using a rodent ventilator (UGO BASILE, model: 7025) (Klawitter et al., 2002 ▶). 

 A mid sternal thoracotomy was performed and a steel cannula inserted through an aortotomy into the aorta and secured by a suture. While in situ, the hearts were immediately perfused using Krebs-Henseleit bicarbonate buffer at a constant pressure of 60- 70 mmHg and a temperature of 37°C. Buffer was bubbled using 95% O2-5% CO2 to attain a pH of 7.4. The heart was quickly excised from the chest and transferred to a Langendorff apparatus while continuously perfused (Klawitter et al., 2002 ▶).

A water-filled latex balloon that was attached to a pressure transducer by a stainless steel needle was inserted through the left atrium into the left ventricle for measuring left ventricular pressure (LVP). The heart was submerged in a jacketed, temperature-controlled glass chamber and allowed to equilibrate for 25-30 minutes. The balloon volume was set to maintain a LV end diastolic pressure (LVEDP) of 5 mmHg. The signal from the pressure transducer was analyzed using a Power Lab system (AD instruments, Australia). Heart rate (HR), coronary flow, left ventricular developed pressure (LVDP, left ventricular end systolic pressure–left ventricular end diastolic pressure), left ventricular end systolic pressure (LVESP), LVEDP, maximum rate of rise (+dp/dt) and maximum rate of fall (-dp/dt) of LVP and rate-pressure product (RPP; product of LVDP and heart rate), as the indices of contraction and relaxation, were measured (Klawitter et al., 2002 ▶). Meanwhile, HR and perfusion pressure were continuously monitored. 

All hearts were perfused for 25-30 minutes to allow stabilization of Left Ventricular Pressure (LVP) and Coronary Perfusion Pressure (CPP) and then (except for the sham group) subjected to 30 minutes of no-flow global ischemia, followed by 60 minutes of reperfusion (Kinugasa et al., 2003 ▶).

**Infarct size measurement**

After the 30-minute ischemia/60-minute reperfusion, the hearts were fixed in 10% formalin to evaluate the infarct size (Cologna et al., 2008). Then, heart samples were sliced (2 mm thickness) and incubated at 37˚C by 1% tri phenyl tetrazoliumchloride (TTC) for 20 minutes. Then, the slices were incubated with 10% formalin for 60 minutes. After that, infarct areas were quantified as the percentage of total area of slice on both sides using image analysis software NIHimagepro.1. (Cologna et al., 2008 ▶).

**Statistical analysis**

The data was analyzed and compared with control values using ANOVA repeated measurement and one-way ANOVA, followed by appropriate post-hoc (LSD) according to the experimental protocols. The level of statistical significance was defined as P<0.05. The data was expressed as mean ± SEM.

## Results

**Effects of standardized Chichorium extract on myocardial function:**

In this study, perfusion pressure was kept stable (60-70±5mmHg) in all groups.

The results showed that heart rate (HR), coronary flow, left ventricular developed pressure (LVDP) and rate of pressure product (RPP) significantly decreased in control group during reperfusion compared to the baseline (before ischemia) (Table 1).

 The hearts which were perfused by *C. intybus *extract (CH1 and CH2) significantly improved post-ischemic contractile function as compared with pre-ischemic status. In CH2 perfused group, HR, LVDP, RPP and coronary flow improved significantly during reperfusion compared with control (P<0.01), the data is shown in Table 1.

First derivative of LVP/dt (+dp/dt), showed a significant decrease in control group as compared to pre-ischemia. *C. intybus *extract significantly increased +dp/dt during reperfusion (Table 1).

**Table 1:** Hemodynamic parameters during baseline and reperfusion periods of ischemia- reperfusion protocol

		**Sham**	**Control**	**CH1**	**CH2**
**LVDP, mmHg**	Baseline	89±10	90±15	69±14	77±8
	Reperfusion		28±6	21±9	47±7^**^
**LVSP, mmHg**	Baseline	90±8	95±15	93±14	83±8
	Reperfusion		35±6	50±3	48±8^*^
**Perfusion Pressure, mmHg**	Baseline	65±4	63±3	57±1	70±10
	Reperfusion		65±3	61±0.5	74±11
**HR, beats/minute**	Baseline	270±11	271±12	240±13	252±13
	Reperfusion		174±16	205±12	217±9^**^
**Coronary Flow, ml/minute**	Baseline	10±0.5	11±1	11±1	11±1
	Reperfusion		4±0.4	7±0.5	7±1^*^
**+dp/dt, mmHg**	Baseline	3000±400	2991±384	3186±319	3117±260
	Reperfusion		606±72	1460±93^**^	1375±270^**^
**RPP, mmHg/minute**	Baseline	28000±5000	23098±2779	21821±3320	19449±2305
	Reperfusion		4319±394	7326±802	10421±737^**^

Effects of *C. intybus *extract (CH1=1mg/ml and CH2=3 mg/ml) on post-ischemic contractile recovery. Time Course of Left Ventricular Developed Pressure (LVDP), Maximal Rate of Pressure Development (+dp/dt), Rate of Pressure Product (RPP), Left Ventricular Systolic Pressure (LVSP), Coronary Flow (CF), Perfusion Pressure and Heart Rate (HR) of non-perfused hearts in Control and *C. intybus *extract-perfused hearts submitted to the ischemia-reperfusion protocol. Higher dose of *C. intybus *extract improved the heart recovery during reperfusion. All groups underwent a 30-minute global ischemia and a 60-minute reperfusion. (Mean ±SEM, n=8, Repeated Measurements followed by LSD,

* P<0.05,

** P<0.01 vs. Control).

**Figure 1:**
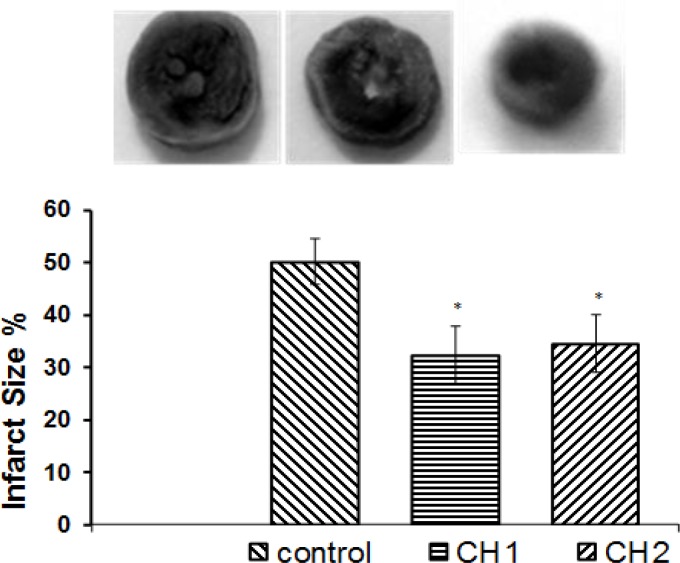
Effect of *C. intybus *extract on myocardial infarct size after ischemia-reperfusion. Hearts were perfused by two different doses of extract (CH1=1mg/ml and CH2=3 mg/ml) while control hearts were perfused by pure perfusion solution. Myocardial infarct size was determined at the end of each experiment as described in the method section (mean±SEM, n=8 in each group, one way ANOVA followed by LSD, *P<0.05 vs. Control

**Figure 2:**
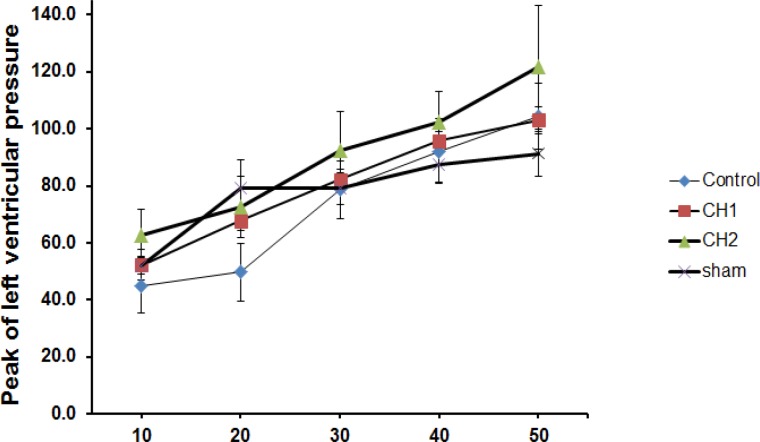
Effect of *C. intybus *extract (CH1=1mg/ml and CH2=3 mg/ml) on peak of left ventricular pressure in different end diastolic pressures after 30-minute ischemia/60-minute reperfusion. There were no significant differences between different groups (mean±SEM, n=8, repeated measurements followed by LSD).

Myocardial infarct size expressed as the percentage of infarct of the entire risk area was 53% for the control group (Figure 1). However, there was a significant reduction in the infarct size for the hearts of the animals receiving *C. intybus* extract 1mg/ml or 3mg/ml (32% and 34%, respectively) (P<0.05). At the end of each experiment, we induced end diastolic pressures (10-50 mmHg) and LVSP were assessed. There were no significant differences between all groups (Figure2).

## Discussion

The results of the current study demonstrated a significant decrease in LVDP, +dp/dt, heart rate, coronary flow and RPP after a 60-minute reperfusion compared with baseline (pre-ischemia) in control group. There was a significant improvement in heart rate, rate of pressure product (RPP), LVDP (LVEDP-LVSP), dp/dt and coronary flow during reperfusion in the hearts perfused by the solvent containing *C. intybus *extract (3mg/ml) compared with control group. Reduction of myocardial size in hearts perfused by *C. intybus *extract was significant compared with control group. 

Since during ischemia ATP production is impaired due to disruption of ETC (electron transport chain), the cell was depleted from high energy phosphates (McCormack et al., 1990 ▶; Territo et al. 2000 ▶). Following these injuries, pyruvate oxidation decreases, and glycolysis, anaerobic metabolism, intracellular acidosis due to lactate and pyruvate accumulation, necrosis and cell death occur (Kim et al., 2006 ▶; Hausenloy et al., 2007 ▶; Yellon and Hausenloy, 2007 ▶). As a result, contractile function declines and Na^+^, Ca^2+^ increases in the cytosol. Oxygen- derived free radicals or oxidative stress play a significant role in a variety of cardiovascular diseases, cardiomyopathy, hypertrophy, atherosclerosis and ischemic heart disease. Since more than two decades ago, the role of reactive oxygen species in many cardiovascular diseases has become increasingly apparent. Under normal conditions, there is a balance between the formation of pro-oxidants (oxygen-free radicals) and the amount of anti-oxidants present. This steady-state condition is interrupted in pathophysiologic conditions because of the excessive production of free radicals and decrement in anti-oxidants or both (Lowenstein, 2007 ▶). 

Previous studies have shown that flavonoid compounds in *Chichorium intybus* cause protective and anti-oxidant effects on liver cells through reducing lipid peroxidation (Madani et al., 2006 ▶). Different mechanisms can be involved in the protective effect on these cells like anti-oxidative effect, lipid peroxidation, detoxification systems stimulator, glutathion ejection, protein synthesis motivation, mast cell stabilization and immunity regulation (Schuppan et al., 1999 ▶; Janbaz et al., 2002 ▶). 

This study showed significant improvement in heart rate, LVDP and RPP in heart of rats perfused by *C. intybus* as compared to control group, during reperfusion. These improvements can be related to the above- mentioned anti-oxidant effects of *C. intybus*. 

It has been reported that *C. intybus *decreases calcium influx from the extra cellular space and induces vasorelaxation in isolated rat aorta strips (Sakurai et al., 2003 ▶). Furthermore, other researchers have reported that vasodilating effects of flavonoids may partly be exerted by scavenging peroxynitrite and therefore preserving tetrahydrobiopterin from oxidation or through inhibiting endothelial NADPH oxidase, which causes the production of O_2_ and promotes the formation of peroxynitrite likely contributes to endothelium dysfunction (Akhlaghi and Bandy, 2009 ▶). Thus, the improvement of coronary flow after reperfusion may be related to this effect. Also, previous studies have shown that a diet containing *C. intybus *can decrease the pH and viscosity in the colon and cecum in rabbits and rats due to inhibition of enzymes’ activity and decrement in the TG, cholesterol and the blood sugar in diabetic rats (Khaksari et al., 2001 ▶; Juskiewicz et al., 2006 ▶; Juskiewicz et al., 2008 ▶). *C. intybus *has inuced a positive effect on coloic apoptosis; therefore, the reduction of infarct size can be attributed to this effect (Hughes and Rowland, 2001 ▶). 

Results of other studies have shown that velocity, frequency and amplitude of spike potential and contractions were higher and stronger than those of control in small intestine in rats fed by diet supplemented with *C. intybus *(Lesniewska et al., 2006 ▶). It was reported that hydroalcholic extract of *C. intybus* has an anti-bacterial effect on Staphylococcus aureus (Ghaderi et al., 2004 ▶). However, a slight and non-significant improvement in post- ischemic left ventricular function may be related to the doses of the extract used in present study. Although we used two different doses of *C. intybus* extract, it is suggested to use several higher doses. 

Considering previous studies in which a significant increase in Na^+^ and K^+^ levels in rats treated by *C. intybus* extract was observed (Rasouli et al., 2000 ▶), it seems that a significant increase in RPP as an index of heart work may be correlated to this effect. This is because when the pH increases in the cell, the Na^+^-H^+^ pump activity also rises to rescue the cell from H^+^ ions. This, in turn, leads to the aggregation of the Na^+^ and weakening of Na^+^-K^+^ pump due to acidosis. After that, the Ca^2+^-Na^+^ exchanger activity is inverted and the high level of Ca^2+^ is accumulated in the cytosol. This effect can impair the mitochondrial metabolism. Therefore, the AMP and other intermediates can be aggregated and cause swelling and rapture of the mitochondrial membrane. As a result, reducing the pH by means of the antioxidative effect of *C. intybus*, we might save the cell (Rasouli et al., 2000 ▶).

Because the LVSP did not change in both the control and Chichorium extract groups when different end diastolic pressures were induced manually in the end of each experiment, we conclude that these doses of extract cannot improve the mechanical recovery during reperfusion. Therefore, more studies with higher doses of this extract and other antioxidants are recommended to be done in the future.

It seems that flavonoid compounds of aqueous extract of *C. intybus* decreases the ischemia-reperfusion injuries, so it can have a protective role in heart function after ischemia. 
